# Parasitic and Vector-Borne Infections in HIV-Positive Patients in Slovakia—Evidence of an Unexpectedly High Occurrence of *Anaplasma phagocytophilum*

**DOI:** 10.3390/pathogens10121557

**Published:** 2021-11-29

**Authors:** Katarína Šimeková, Ľubomír Soják, Bronislava Víchová, Lenka Balogová, Júlia Jarošová, Daniela Antolová

**Affiliations:** 1 Clinic of Infectology and Travel Medicine, Jessenius Faculty of Medicine and University Hospital in Martin, Comenius University Bratislava, Kollárova 2, 036 01 Martin, Slovakia; katarina.simekova@uniba.sk; 2 Department of Infectology and Geographical Medicine, Center for Treatment of HIV/AIDS Patients, Academic L. Derer’s University Hospital, Limbová 5, 833 05 Bratislava, Slovakia; lubosojak@centrum.sk; 3 Laboratory of Human Parasitology, Institute of Parasitology of Slovak Academy of Sciences, Hlinkova 3, 040 01 Košice, Slovakia; vichova@saske.sk (B.V.); jarosova@saske.sk (J.J.); 4 Clinic of Infectology and Travel Medicine, University Hospital of L. Pasteur, Rastislavova 43, 040 01 Košice, Slovakia; lenka.balogova@unlp.sk

**Keywords:** HIV infection, *Toxoplasma gondii*, *Anaplasma phagocytophilum*, human granulocytic anaplasmosis, *Borrelia burgdorferi* sensu lato, CD4 T-lymphocytes

## Abstract

In HIV (human immunodeficiency virus) infected people, the immunodeficiency caused by a reduced level of CD4 (cluster of differentiation 4) T-lymphocytes increases the risk of infectious diseases. Additionally, in individuals with immunologically compromising conditions, tick-borne or some parasitic pathogens may cause chronic, debilitating opportunistic infections and even death. The study aimed at determining the IgG seropositivity of HIV-infected patients to *Toxoplasma gondii*, *Toxocara* spp., *Echinococcus multilocularis*, and *E. granulosus* s.l. and performing the molecular identification of *T. gondii* and some tick-borne pathogens, namely, *Borrelia* spp., *Babesia* spp., *Anaplasma phagocytophilum*, *Rickettsia* spp., and *Bartonella* spp. Out of 89 HIV-positive patients, specific IgG antibodies to *T. gondii* were detected in 17 (19.1%) and to *Borrelia* spp. in 12 (13.5%) individuals. Seropositivity to *Toxocara* spp., *E. multilocularis,* and *E. granulosus* s.l. was not recorded. Molecular approaches showed positivity to *T. gondii* in two (2.2%) patients, and 11 (12.4%) individuals had positive PCR signal for the *msp2* gene of *A. phagocytophilum*. Relatively high prevalence of *A. phagocytophilum* in HIV-positive patients suggests that these people are more susceptible to some vector-borne pathogens. The presence of opportunistic infections may pose a health risk for patients with weakened immune systems, and should not be neglected during the regular monitoring of the patient’s health status.

## 1. Introduction

In human immunodeficiency virus (HIV) infected people, the immunodeficiency caused by reduced levels of CD4 (cluster of differentiation 4) T-lymphocytes, destroyed due to constant multiplication of the virus, increases the risk of infectious diseases [1]. The gradual decrease in CD4 T-lymphocyte cells (below 200 cells/µL) ultimately results in a loss of control over immune response and the development of various opportunistic infections [1,2,3].

The introduction of antiretroviral drugs and effective regimens of highly active antiretroviral therapy (HAART) have significantly improved the prognosis for HIV-infected, and HIV-related mortality rates and the incidence of AIDS in the developed countries have declined substantially [4]. Consequently, HIV-infected individuals have greater opportunities for an active lifestyle and outdoor activities, which, on the other hand, increases the risk of transmission of soil, tick-borne, or other pathogens [5,6]. Additionally, in individuals with immunologically compromising conditions, tick-borne, or some parasitic pathogens may cause chronic, debilitating opportunistic infections and even death [6,7,8].

One of the major opportunistic infections afflicting patients with advanced HIV infection is toxoplasmosis, caused by the protozoan *Toxoplasma gondii*. The primary infection in immunocompetent individuals is typically asymptomatic or accompanied with mild symptoms, and usually followed by a lifelong latent infection. Nevertheless, from the state of latency, *T. gondii* infection may be reactivated as a result of immune disorders [9]. Similarly, uncontrolled proliferation of *Echinococcus multilocularis* metacestode leading to rapidly progressing alveolar echinococcosis has been described in individuals with impaired immunity caused by AIDS, other immunodeficiencies, or immunosuppressing therapy [10,11]. 

In Europe, several tick species are widely distributed and prevalent and can serve as vectors of tick-borne pathogens, including *Borrelia burgdorferi* sensu lato (s.l.), *Anaplasma phagocytophilum*, *Babesia* spp., *Bartonella* spp., and *Rickettsia* spp.

So far, several cases of *B. burgdorferi* s.l. [7,12,13] and individual *Babesia*, *Rickettsia,* and *Anaplasma* infections, mostly in the form of single case presentations, have been reported in HIV-infected people [5,14,15]. Seropositivity to tick-borne pathogens, namely, *B. burgdorferi* s.l., *Babesia microti*, *A. phagocytophilum*, *Ehrlichia* spp., *Bartonella henselae,* and *Rickettsia* spp., was recently reported in HIV-infected patients from Poland [6], suggesting more frequent contact with ticks and consequently a higher risk of tick-borne infection than had been previously assumed.

The study was aimed at determining the IgG seropositivity of HIV-infected patients to selected parasites, namely, *T. gondii*, *Toxocara* spp., *E. multilocularis*, and *E. granulosus* s.l., and performing the molecular identification of some tick-borne pathogens, *Borrelia* spp., *Babesia* spp., *A. phagocytophilum*, *Rickettsia* spp., and *Bartonella* spp. Since the CD4 T-lymphocyte count is an important parameter of HIV disease progression, the correlation between the prevalence of infection and the number of CD4 lymphocytes was also assessed. 

## 2. Results

Out of 89 monitored HIV-positive patients, specific IgG antibodies only to *T. gondii* and *Borrelia* spp. were detected, with 17 (19.1%) and 12 (13.5%) positive individuals, respectively. No patients were found to be seropositive to *Toxocara* spp., *E. multilocularis,* and *E. granulosus* s.l. (Table 1).

Molecular approaches showed positivity for *A. phagocytophilum* and *T. gondii*. Out of all patients, 11 (12.4%) had a positive PCR signal for the *msp2* gene of *A. phagocytophilum* (Table 1). Nucleotide sequences obtained were added to the GenBank database and are available under the following accession numbers: MZ945428–MZ945438. 

Positive patients came from western, central, and eastern Slovakia; it means that the positivity was recorded throughout the country. Though the blood samples were collected in autumn and winter of 2020, and then in the spring and early summer of 2021, the positivity was recorded only in patients sampled in May and June 2021. Positive patients did not report any clinical signs of infection.

A positive PCR signal for the fragment of *TGR1E* gene of *T. gondii* was recorded in two (2.2%) patients, one being seropositive and one seronegative when examined by ELISA. On the other hand, none of the patients, either seropositive or seronegative, were PCR positive to *Borrelia* spp. No PCR positivity was recorded also in the case of *Babesia* spp., *Bartonella* spp., and *Rickettsia* spp. (Table 1). 

The average number of CD4 T-lymphocytes in all engaged patients was 663.2 ± 257.0 and ranged from 77 to 1150/μL of blood. The average numbers of CD4 cells in patients seropositive or PCR positive to *T. gondii*, *Borrelia* spp., and *A. phagocytophilum* were lower than those in negative individuals (Table 2). However, the only statistically significant result was the difference between patients who were seropositive and seronegative for *T. gondii*, with 541.7 ± 320.7 and 691.9 ± 233.1 CD4 T-lymphocytes/μL of blood for seropositive and seronegative, respectively (Table 2).

In both patients molecularly positive to *T. gondii* (one seropositive and one seronegative one), the number of CD4 T-lymphocytes was close to 200 per microliter of blood (224 and 226/μL, respectively).

In some patients, mixed infections occurred, e.g. seropositivity/PCR positivity to two or three pathogens, with the combination of *T. gondii* seropositivity and *A. phagocytophilum* PCR positivity being the most common (Table 3).

## 3. Discussion

In patients with HIV infection, immunodeficiency significantly increases the risk of diseases caused by pathogens that expand as a consequence of the reduced level of CD4 T-lymphocytes, since pathogen load is usually controlled by humoral and cellular immune responses [2]. So far, the majority of epidemiological studies of parasitic and tick-borne pathogens have focused on people of higher risk of infection or on inhabitants of endemic regions whose immunological status was not impaired. However, the risk of developing serious complications from *T. gondii* infection in HIV-infected people is well known, and published reports on some other parasitic (e.g., echinococcosis) and tick-borne infections have shown that such diseases in immunocompromised patients can also be more severe, prolonged, or even fatal [6,7,10,16]. 

Our study focused on IgG seropositivity and molecular identification of selected pathogens in HIV-positive patients in Slovakia. Out of 89 serum samples, specific IgG antibodies to *T. gondii* were detected in 19.1% of the samples and 13.5% tested positive for antibodies to *Borrelia* spp. In addition to this, 12.4% of samples were PCR positive to *A. phagocytophilum. A. phygocytophilum* is a Gram-negative, obligate intracellular bacterium transmitted by *Ixodes ricinus* and *I. trianguliceps* (within sylvatic cycle) ticks [17,18]. Human granulocytic anaplasmosis (HGA) is an emerging infectious disease, which is observed after the bacteria are inoculated into the host, following a tick bite. The course of infection might vary from subclinical or mild to severe, with the need of hospitalization or even the death of patient, and the clinical manifestation includes nonspecific flu-like symptoms, such as fever, myalgia, headache, thrombocytopenia, leukopenia, anemia, and hepatic injury [19,20,21]. There are only a few studies dealing with *A. phagocytophilum* in HIV-positive patients. The very first case of HGA in an HIV-positive was reported by Welc-Falęciak et al. [5] in Poland, with one patient identified as PCR positive for *A. phagocytophilum* within the retrospective study of 148 HIV-positive patients with no history of tick bite and no clinical manifestation characteristic for tick-borne diseases. Later, the seroprevalence study on 227 HIV-positive patients and 199 blood donors in Poland reported by Pawelczyk and colleagues [6] showed 2.6% vs. 3.0% IgM seropositivity, and 2.6% vs. 1.5% IgG seropositivity, suggesting no significant differences between HIV-positive and healthy people.

In the presented study, out of 89 patients, 11 (12.4%) were PCR positive to *A. phagocytophilum*, suggesting higher susceptibility of immunocompromised patients to infection. Positive patients came from the entire territory of the country, from western, central, and eastern Slovakia. Though the blood samples were collected in autumn and winter of 2020 and then in the spring and early summer of 2021, the positivity was recorded only in patients sampled in May and June 2021, which corresponds with the activity peak of questing Ixodid ticks. Patients that presented positive did not report any signs of the infection; however, HIV-infected patients often suffer from symptoms connected with HIV that can overlap with nonspecific signs of other infections. The number of CD4 T-lymphocytes was not significantly lower than in negative patients; nevertheless, possible severe complications in HIV-positive people especially in the stage of advanced immunosuppression cannot be excluded.

In general, cases with the confirmed presence of *A. phagocytophilum* DNA in blood samples are rare and usually reported as single/few cases presentations. Single patients with clinical manifestation and positive PCR were reported from South Korea in 2013 and 2015 [22,23]. Later, between 2015 and 2017, a study on 380 patients with a tick bite or outdoor activity history and with febrile syndrome showed 14 PCR-positive cases [24]. In Taiwan, an examination of 274 blood samples from patients suspected of having scrub typhus revealed PCR positivity in two samples [25]. A case of HGA imported from the USA to Austria and a case with fatal outcome of the disease associated with macrophage activation syndrome in Greece were diagnosed in 2015 [21,26]. In the USA, HGA was also molecularly confirmed in a patient with nontraumatic splenic rupture [27]. In Slovakia, the first case of *A. phagocytophilum* infection was molecularly confirmed in a patient from the northern part of the country in 2010 [28]. Recently, two more cases have been published, representing 1.7% positivity after PCR examination of 118 blood samples collected between 2011 and 2020 [29]. Moreover, 170 blood samples of suspected patients (with a tick bite history and nonspecific clinical signs) have been examined for *A. phagocytophilum* at the Institute of Parasitology SAS since 2019, and only 4 (2.4%) tested PCR positive [30]. Important is the fact that the number of PCR positives recorded in the presented study (12.4%) was higher than the data from seropositive people with the tick bite history and/or clinical signs of tick-borne infections (however, with unknown HIV status). Seropositivity to *A. phagocytophilum* in people is usually reported in areas with the presence of the pathogen and competent vectors, and the seropositivity rates vary depending on the frequency of activities predisposing to or predicting tick exposures and bites [19,31]. A recently published systematic review and meta-analysis showed pooled 8.4% *A. phagocytophilum* seroprevalence in 28,927 individuals examined within 56 studies performed between 1994 and 2008. The seropositivity ranged from 0.0% to 37.3% and pooled seroprevalence was highest in a high-risk population (13.8%) and lowest in a healthy population (5.0%) [32]. In Slovakia, the study on 76 sera collected in the period 2002–2005 from patients with a tick bite history and symptoms resembling Lyme borreliosis showed 25% seropositivity [33]. Later, analysis of 214 serum samples from patients suspected to be infected with Lyme borreliosis revealed 7.0% seropositive to *A. phagocytophilum* [34].

*Borrelia burgdorferi* s.l. complex comprises the etiological agent of Lyme disease, the most common tick-borne infection in Europe and the USA. Clinical signs of infection include erythema migrans, and later, when the spirochetes penetrate into body tissues, Lyme borreliosis can manifest as arthritis, skin disorders, or some neurological manifestations. In Europe, at least five species are considered to be pathogenic for humans (*B. afzelii*, *B. garinii*, *B. burgdorferi*, *B. spielmanii*, and *B. bavariensis*), resulting in a higher variety of clinical symptoms that can influence the diagnosis of disease [35].

PCR on blood samples has not been shown to be of diagnostic relevance and is not recommended for clinical diagnosis of Lyme disease due to time limitation on a short period of acute infection, a low number of spirochetes in blood, or transient spirochetemia [35]. However, this might be different in immunocompromised patients. Therefore, both molecular detection of intergenic spacer (*IgS*) fragment of *B. burgdorferi* s.l. and serological approach were used in the study. While anti-*Borrelia* IgG antibodies were recorded in 12 (13.5%) patients, none of the 89 examined individuals were PCR positive. The data on seropositivity of borreliosis among HIV-infected people are rare. In the study of Pawelczyk et al. [6], IgG seropositivity among 227 HIV-positive patients was slightly lower (4.8%) than seropositivity of 199 healthy blood donors (5.0%). In a healthy population, seroprevalence data greatly vary based on the frequency of activities predisposing tick infestation. In Belgium, 2.6% and 2.9% seropositivity of urban and rural blood donors was reported [36], while as many as 21.6% of forests workers were positive [37]. The study from Serbia showed 11.76% seroprevalence of *B. burgdorferi* in a group of forestry workers, 17.14% in a group of soldiers, and 8.57% in healthy blood donors [38]. In Germany, the study on 6945 healthy adults revealed significantly higher seroprevalence among men (13.0%) than among women (5.8%), and seropositivity increased by age among both genders [39]. The locality-based differences confirmed the study of Zajac et al. [40], in which the prevalence varied between 18.2% and 50.7%, suggesting a focal occurrence of borreliosis. In Slovakia, a seroprevalence study on 439 patients suspected of Lyme borreliosis performed 20 years ago recorded 9.6% IgG positivity [41].

*Borrelia* spp. seropositivity, as well as the positivity to *A. phagocytophilum*, detected in the presented study suggests previous tick infestation and consequent exposure to tick-borne pathogens in HIV-infected patients; however, no correlation between the positivity rate and the number of CD4 T-lymphocytes was recorded for both pathogens.

The overall seroprevalence of *T. gondii* IgG antibodies recorded in the presented study was 19.1%, with a significantly lower number of CD4 T-lymphocytes in seropositive than in seronegative patients. Moreover, the DNA of the parasite was detected in two patients, one being seropositive and one seronegative to *T. gondii*. The number of CD4 T-cells in both patients was close to 200 per microliter of blood, suggesting the progression of immunodeficiency and possible reactivation of infection in seropositive patient. In general, people who acquire HIV infection and who were not previously infected are more likely to develop severe primary toxoplasmosis, while people who were infected before they become immunosuppressed are at risk for developing a reactivation of the disease. A HIV-infected person who has reactivated *Toxoplasma* infection can have symptoms that include fever, confusion, headache, seizures, nausea, and poor coordination [42]. Therefore, although *T. gondii*-infected HIV-positive patients do not complain of these signs (as in the presented study), further complications cannot be excluded and they should be under proper medical control.

The seropositivity rate recorded in this study was lower than that in HIV-positive patients from Slovakia examined in 2019 (34.4%) [43], and also lower than the pooled prevalence of latent toxoplasmosis calculated for Europe (41.1%) in the systematic review of studies published by Rostami et al. [44]. Since the seropositivity to *T. gondii* greatly varies among regions and correlates with the socio-economic conditions and the presence of infection risk factors [44], the reported seropositivity in the presented study could be influenced by such aspects.

Alveolar and cystic echinococcosis are severe parasitic infections that can significantly alter the health or even cause the death of the patient. In humans, who are unsuitable hosts for *E. multilocularis*, repeated or long-term exposure is required to become infected [45]. In the presented study, none of the monitored patients were seropositive to *Echinococcus* spp. We can suppose that HIV-infected people, aware of their worsened immune status, are more careful and follow hygiene measures during outdoor activities; thus, they are not at higher risk of infection. However, once the infection is established, HIV infection can accelerate the proliferation of metacestode tissues and the progress of the disease [10,45].

## 4. Materials and Methods

### 4.1. Material

The group analyzed comprised 89 patients monitored at three specialized outpatient clinics situated in three different areas of Slovakia, in western, central, and eastern Slovakia. Thus, samples from patients coming from three geographically different areas of the country were obtained. The blood samples and patient data were collected during the regular control visits from September to December 2020 and from March to July 2021.

The study group was composed of 84 men and 5 women. Their ages varied between 20 and 72 years, and the average age was 39.8 years. All patients had been on antiretroviral therapy for at least half a year.

### 4.2. Serological Methods

In-house indirect enzyme-linked immunosorbent assay (ELISA) was used to detect antibodies to *Toxocara* spp., *E. multilocularis*, and *E. granulosus* s.l. in the patient’s sera. Specific IgG antibodies to *Toxocara* spp. were determined by ELISA described previously [46]. Antibodies to a somatic antigen of *E. multilocularis* and antigen B of *E. granulosus* s.l. were detected according to the ELISA described by Fecková et al. [47]. 

The presence of IgG anti-*T. gondii* antibodies was determined using commercial EIA *Toxoplasma* IgG (Test-Line Ltd., Clinical Diagnostics, Brno, Czech Republic) diagnostic kits, and IgG antibodies to *Borrelia* spp. were detected by using a commercial *Borrelia* IgG ELISA Recombinant Antigen (Biomedica, Wien, Austria) test kit. Microwell strips of the test are coated with recombinant antigens of *B. burgdorferi* sensu stricto, *B. garinii,* and *B. afzelii*. 

The number of CD4 T-lymphocytes in blood samples was analyzed using the standard flow cytometry and is indicated in the absolute number of cells per microliter (μL) of blood. 

### 4.3. Molecular Methods

Genomic DNA from blood samples was extracted using a Thermo Scientific GeneJET Genomic DNA Purification Kit (Thermo Fisher Scientific Baltics UAB, Vilnius, Lithuania). Subsequently, PCR assays targeting the 191 bp fragment of *TGR1E* gene of *T. gondii* as described by Lamoril et al. [48], *msp2* gene of *A. phagocytophilum* (334 bp) described by Massung et al. [49], *18S rRNA*-encoding gene fragment of *Babesia* spp. (411–452 bp) based on the description of Casati et al. [50], a noncoding RNA (*ssrA*) gene of *Bartonella* spp. (250–300 bp), and intergenic spacer (*IgS*) of members of *B. burgdorferi* sensu lato complex with a length of 222–255 bp described by Derdáková et al. [51] were performed. An approximately 338 bp long fragment of *gltA* gene of *Rickettsia* spp. was amplified by nested PCR as published by Regnery et al. and Choi et al. [52,53]. 

The results of PCR assays were visualized after gel electrophoresis under UV light, using a UV transilluminator. Positive PCR products were purified using the Nucleospin Extract II kit (Macherey Nagel, Düren, Germany) and sent for bidirectional sequencing using the same primers as used for the PCR assays. 

The obtained sequences were compared with GenBank entries using Basic Local Alignment Search Tool (BLAST) [54] for determining the sequence identities and species matches. 

### 4.4. Statistical Analyses

Statistical differences between the numbers of CD4 T-lymphocytes were evaluated using the one-way ANOVA, which allowed the comparison of continuous variables in two groups of patients, with a value of *p* < 0.05 considered significant. Prevalence/seroprevalence of infection is presented as the relative frequency with a 95% confidence interval (95% CI).

## 5. Conclusions

The study confirmed the seropositivity of HIV-infected patients to *T. gondii* a *Borrelia* spp. and molecular positivity to *A. phagocytophilum* and *T. gondii*. Relatively high prevalence of *A. phagocytophilum* in HIV-positive patients suggests that these people are more prone to become infected with serious vector-borne pathogens. However, further studies are necessary to clarify the correlation between immunodeficiency in HIV-positive people and *A. phagocytophilum* infection. The presence of opportunistic infections in patients with weakened immune system may pose a real health risk for this part of the population, and it certainly should not be neglected during the regular monitoring of the patient’s health status. A tick bite history should be included in the anamnesis of these patients to ensure early intervention in case of health complications due to associated vector-borne infections.

## Figures and Tables

**Table 1:** Seropositivity/PCR positivity of HIV-positive patients examined for selected parasitic and tick-borne pathogens.

IgG Seropositivity	Positive (n = 89)	Prevalence (%)	95% CI
*Toxoplasma gondii*	17	19.1	11.5–28.8
*Toxocara* spp.	0	0.0	0.0–4.1
*Echinococcus multilocularis*	0	0.0	0.0–4.1
*Echinococcus granulosus* s.l.	0	0.0	0.0–4.1
*Borrelia* spp.	12	13.5	7.1–22.3
**PCR positivity (gene)**			
*Toxoplasma gondii* (*TGR1E*)	2 *	2.2	0.3–7.9
*Anaplasma phagocytophilum* (*msp2*)	11	12.4	6.3–21.0
*Babesia* spp. (*18S RNA*)	0	0.0	0.0–4.1
*Bartonella* spp. (*ssrA*)	0	0.0	0.0–4.1
*Rickettsia* spp. (*gltA*)	0	0.0	0.0–4.1
*Borrelia* spp. (*IgS*)	0	0.0	0.0–4.1

n, number of examined; 95% CI, 95% confidence interval; * one seropositive, one seronegative.

**Table 2:** Average number of CD4 T-lymphocytes in HIV-infected patients positive/negative to selected parasitic and tick-borne pathogens.

Pathogen	CD4 T-lymphocytes/μL ± SD in Positive Patients	CD4 T-lymphocytes/μL ± SD in Negative Patients	*p*-Value
*Toxoplasma gondii* Seropositivity	541.7 ± 320.7	691.9 ± 233.1	0.03
*Borrelia* spp. Seropositivity	608.4 ± 362.2	671.7 ± 238.6	0.43
*Anaplasma phagocytophilum* PCR positivity	621.1 ± 470.8	669.1 ± 215.4	0.56
*Toxoplasma gondii* PCR positivity	225.0 ± 1.4	673.3 ± 251.0	nc

SD, standard deviation; nc, not calculated.

**Table 3:** Occurrence of mixed infection/seropositivity in HIV-positive patients.

Mixed Infection/Seropositivity	No. of Patients (%)
*Toxoplasma gondii* sero/PCR positivity *Borrelia* spp. seropositivity	3 * (3.4)
*Toxoplasma gondii* seropositivity *Anaplasma phagocytophilum* PCR positivity	5 (5.6)
*Borrelia* spp. seropositivity *Anaplasma phagocytophilum* PCR positivity	2 (2.2)
*Toxoplasma gondii* seropositivity *Borrelia* spp. seropositivity *Anaplasma phagocytophilum* PCR positivity	1 (1.1)

* one patient was PCR positive, but seronegative to *T. gondii.*

## Data Availability

Data sharing is not applicable to this article.
